# Characterization of Multidrug Resistant Extended-Spectrum Beta-Lactamase-Producing *Escherichia coli* among Uropathogens of Pediatrics in North of Iran

**DOI:** 10.1155/2015/309478

**Published:** 2015-05-03

**Authors:** Mohammad Sadegh Rezai, Ebrahim Salehifar, Alireza Rafiei, Taimour Langaee, Mohammadreza Rafati, Kheironesa Shafahi, Gohar Eslami

**Affiliations:** ^1^Nosocomial Infection Research Center, Mazandaran University of Medical Sciences, Buali Sina Hospital, Sari, Iran; ^2^Department of Clinical Pharmacy, Faculty of Pharmacy, Thalassemia Research Center, Mazandaran University of Medical Sciences, Sari, Iran; ^3^Molecular and Cell Biology Research Center, Department of Immunology, Faculty of Medicine, Mazandaran University of Medical Sciences, Sari, Iran; ^4^Department of Pharmacotherapy and Translational Research, University of Florida, Box 100486, Gainesville, FL 32610-0486, USA; ^5^Fatemeh Zahra Hospital, Mazandaran University of Medical Sciences, Sari, Iran; ^6^Department of Clinical Pharmacy, Faculty of Pharmacy, Mazandaran University of Medical Sciences, Km 18 Khazarabad Road, Khazar Square, Sari, Mazandaran 48471-16548, Iran

## Abstract

*Escherichia coli* remains as one of the most important bacteria causing infections in pediatrics and producing extended-spectrum beta-lactamases (ESBLs) making them resistant to beta-lactam antibiotics. In this study we aimed to genotype ESBL-producing *E. coli* isolates from pediatric patients for ESBL genes and determine their association with antimicrobial resistance. One hundred of the *E. coli* isolates were initially considered ESBL producing based on their MIC results. These isolates were then tested by polymerase chain reaction (PCR) for the presence or absence of *CTX*, *TEM*, *SHV*, *GES*, and *VEB* beta-lactamase genes. About 30.5% of isolated *E. coli* was ESBL-producing strain. The *TEM* gene was the most prevalent (49%) followed by *SHV* (44%), *CTX* (28%), *VEB* (8%), and *GES* (0%) genes. The ESBL-producing *E. coli* isolates were susceptible to carbapenems (66%) and amikacin (58%) and showed high resistance to cefixime (99%), colistin (82%), and ciprofloxacin (76%). In conclusion, carbapenems were the most effective antibiotics against ESBl-producing *E. coli* in urinary tract infection in North of Iran. The most prevalent gene is the TEM-type, but the other resistant genes and their antimicrobial resistance are on the rise.

## 1. Introduction

Multidrug resistant Gram-negative bacilli have been increasingly responsible for life-threatening infections all over the world [1, 2]. The extended-spectrum beta-lactamases (ESBLs) are class A plasmid mediated enzymes that hydrolyze oxyiminocephalosporin and monobactam antibiotics but are inhibited by clavulanic acid in vitro [3, 4]. Bacteria harboring ESBLs confer significant resistance to penicillin, narrow and extended-spectrum cephalosporin, and aztreonam antibiotics. They also frequently show resistance to aminoglycosides, trimethoprim/sulfamethoxazole, and quinolones [5, 6]. Different types of ESBLs have been found in different countries. The* TEM* and* SHV* types were first reported from* Klebsiella pneumoniae* in Western Europe [7]. The* VEB* was first found in a single isolate of* E. coli* in Vietnam [5]. But recently, the* CTX* type (mainly cefotaximases) is being detected with increasing frequency, particularly in ESBL-producing* E. coli* [8]. The GES-5 gene was first detected on plasmid in* Escherichia coli* from Greece in 2004 and later it was isolated from* Klebsiella pneumoniae*,* Enterobacter cloacae*, and* Pseudomonas aeruginosa* [9]. The* CTX*-*M* enzymes are much more active against cefotaxime and ceftriaxone than against ceftazidime [3, 10, 11].

Genotypes of ESBLs producing isolates may be associated with the antibiotic resistance pattern, as it has been reported previously that the presence of* CTX-M* gene has been associated with the resistance to fluoroquinolones, aminoglycosides, and cotrimoxazole [11].

Extensive use of expanded-spectrum antibiotics is one of the most important factors associated with high resistance to antibiotics and high prevalence of ESBLs [12–14].

Since increasing the rate of multidrug resistant ESBL in North of Iran and antibiotics use varies in different regions and can cause variation in the prevalence of ESBL genotypes, we decided to determine the genotype of ESBL-producing* E. coli* in urinary tract infection regarding the* CTX*,* TEM*,* SHV*,* GES*, and* VEB* genes and their antimicrobial resistance in the North of Iran.

## 2. Material and Methods 

### 2.1. Urine Collection and Bacterial Isolation

Urine samples were collected from pediatric patients during a 6-month period at Buali Sina Hospital (a tertiary referral pediatric hospital) in the North of Iran. Urine samples were collected by either midstream clean catch, suprapubic bladder aspiration (SPA), or transurethral bladder catheterization (TUBC) [15]. The samples were inoculated on 5% blood agar and MacConkey's agar and the* E. coli* isolates were identified by using standard methods.

### 2.2. Antimicrobial Susceptibility Testing

Antibiotic susceptibility was determined by the Kirby-Bauer disc diffusion test on Mueller Hinton agar plate and the results were evaluated according to the Clinical Laboratory Standards Institute (CLSI) guideline 2011 [16].

### 2.3. Detection of ESBL-Producing* E. coli* by MIC

The* E. coli* isolates were considered potential carrier of ESBL enzyme when they showed resistance to cefotaxime and ceftriaxone. The MIC (minimum inhibitory concentration) of fourteen antibiotics including ceftazidime, ceftriaxone, cefotaxime, ceftizoxime, cefepime, cefixime, gentamicin, amikacin, meropenem, imipenem, ciprofloxacin, cotrimoxazole, colistin, and piperacillin/tazobactam (Sigma Chemical Co., Germany) for resistant* E. coli* strains was determined by the agar dilution method [3]. The MIC was determined on Mueller Hinton agar with twofold dilutions of antibiotics concentration (from 0.5 *μ*g/mL to 256 *μ*g/mL and 10 *μ*L of microbial suspension). Microbial growth was observed and documented after 24 hours of incubation at 35°C. The result was reported according to CLSI 2011 guidelines and divided into three categories: resistant, intermediate, and susceptible. The ESBL-producing* E. coli* isolates were considered resistant to both cefotaxime and ceftazidime if their MIC was ≥2 *μ*g/mL in accordance with CLSI criteria [17].

### 2.4. DNA Isolation and Genotyping

A single colony from each ESBL-producing isolate was transferred into 100 *μ*L of distilled water and the bacterial DNA was extracted by using a commercial DNA extraction kit (RTA, Ankara, Turkey). Bacterial genes associated with antimicrobial resistance phenotypes were detected by PCR amplification of target genes by using specific PCR primers (Table 1) in Eppendorf thermal cycler (Eppendorf, Germany). Table 1 shows primer sequences and specific thermal profile from* TEM*,* SHV*,* CTX*,* VEB*, and* GES* genes. PCR mixtures were prepared by using 5 *μ*L template DNA, 12.5 *μ*L PCR master mix; 1 × PCR buffer [Tris-Cl, KCl, (NH4)_2_SO_4_, 1.5 mM MgCl_2_] (pH 8.7), 200 *μ*M dNTP, and 1 *μ*L of each 10 pM primer and 0.5 U Taq DNA polymerase (Takara, Kyoto, Japan) in a final volume of 25 *μ*L. In all experiments, the following reference strains were used as positive controls:* K. pneumoniae* 7881 (*CTX*-*M*),* K. pneumoniae* 7881 strain (containing* TEM* and* SHV*),* P. aeruginosa* ATCC 27853 (VEB-1), and* K. pneumoniae* (*GES*) which was kindly provided by Professor P. Nordmann CHU Bicetre, France. A non-ESBL-producing strain (*E. coli* ATCC 25922) was used as a negative control.

## 3. Results

### 3.1. ESBL-Producing* E. coli*

Of 327 uropathogen* E. coli* isolates, one hundred (30.5%) were positive for extended-spectrum beta-lactamases. The ESBL isolates showed highest susceptibility to carbapenems (66%) and amikacin (58%) which is in accordance with 2011 CLSI criteria for MIC test (Table 2). The highest rate of resistance was observed for the following antibiotics: cefixime (99%), colistin (82%), and ciprofloxacin (76%).

### 3.2. Prevalence of ESBL Genes

**Figure 1:**
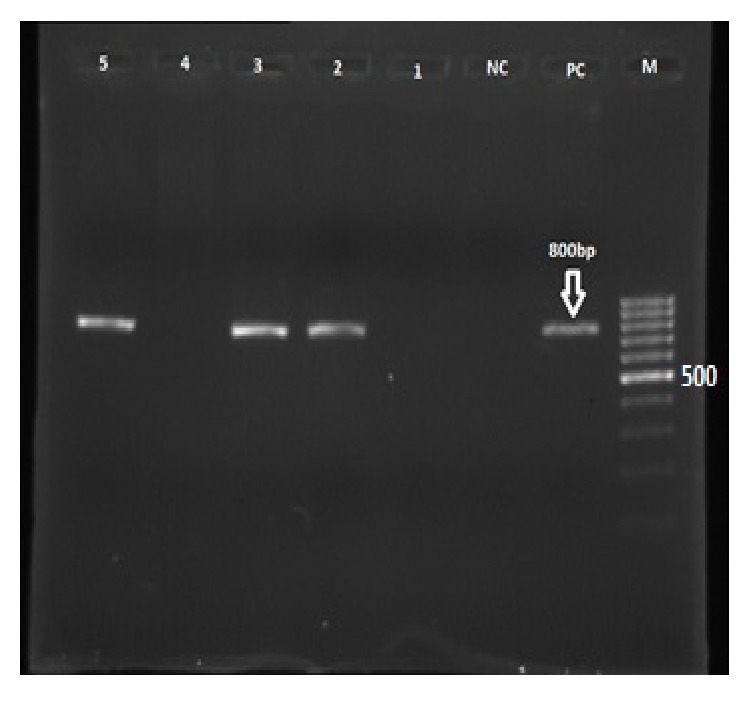
Agarose gel showing the 800 bp PCR fragments band for* TEM* gene from ESBL-producing* E. coli* isolates. Lanes: M: molecular weight marker (100 bp); PC:* K. pneumoniae* 7881 (positive control); NC:* E. coli* ATCC 25922 (negative control); 2, 3, and 5:* TEM* positive clinical samples; 1 and 4:* TEM* negative clinical samples.

**Figure 2:**
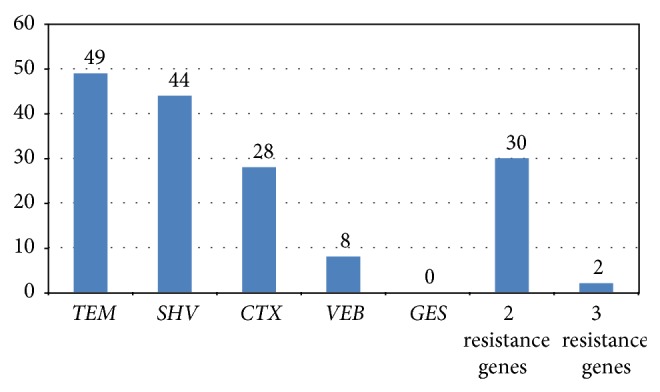
Distribution of* TEM*,* CTX*,* SHV*,* GES*, and* VEB* genes in ESBL-producing* E. coli* isolates.

The results of ESBL genotyping are shown in Figure 1. The* TEM* gene was the most prevalent (49%) followed by* SHV* (44%),* CTX* (28%),* VEB* (8%), and* GES* (0%) genes. None of our isolates carried the* GES* gene. About 12% of ESBL isolates were shown to have both* TEM* and* CTX-M* genes. Overall, 30% of isolates carried 2 resistant genes (Figure 2).

### 3.3. Association of Antimicrobial Resistance with Resistant Genes

Table 3 shows the presence or absence of resistant genes, susceptibility, and resistance to different antimicrobial agents. In most cases, there were not any significant differences regarding presence or absence of genes expression. Interestingly, higher resistance to cefotaxime, amikacin, and ceftriaxone was found in* TEM* negative group (*P* = 0.04, *P* = 0.008, and *P* = 0.02, resp.).

Resistance to cotrimoxazole, imipenem, amikacin, and third generation cephalosporins was observed more in* CTX-M* positive isolates than in* CTX-M* negative isolates. The presence of* VEB* gene was associated with higher resistance to carbapenems, gentamicin, and third generation cephalosporins. The presence of* VEB* gene was significantly associated with resistance to ceftazidime (*P* = 0.05). The presence of* SHV* gene was also associated with aminoglycosides and ciprofloxacin resistance. Our results also showed a correlation between the presence of resistant genes and high rate of resistance to cefixime, colistin, and cefepime.

## 4. Discussion

In this study, attempt has been made to genotype the ESBL-producing* E. coli* isolates from pediatric patients for* CTX*,* TEM*,* SHV*,* GES*, and* VEB* genes and determine their association with antimicrobial resistance. The high prevalence of ESBL-producing* E. coli* (30.5%) and their high level of resistance to broad spectrum antimicrobial agents in ESBL-producing* E. coli* (e.g., 34% resistance to carbapenems) are reported in this study. In addition, our study also highlights an association between the presence of* SHV* gene and resistance to aminoglycosides and fluoroquinolones antibiotics.

The prevalence of ESBL-producing* E. coli* isolates varies in different parts of the world and even among different hospitals within a country. The rate of prevalence in our center was about 30.5% which is close to the results reported by other studies in different regions of Iran [18, 23–25]. The rates of ESBL-producing* E. coli* were lower in other countries such as India (27%), Lebanon (13.3%), Korea (9.2%), and turkey (17%) [26, 27].

In addition to beta-lactam antibiotics, ESBLs producing isolates are also resistant to other antimicrobial agents, such as aminoglycosides, tetracycline, and trimethoprim/sulfamethoxazole [28]. The isolates showed high resistance to amikacin (34%), colistin (82%), and trimethoprim/sulfamethoxazole (65%) in our study. The study by Babypadmini and Appalaraju reported 74% resistance to trimethoprim/sulfamethoxazole and 91.6% resistance to fluoroquinolone in ESBL-producing* E. coli* pathogens by disk diffusion method [29], which is much higher than our results (65% resistance to trimethoprim/sulfamethoxazole and 76% resistance to fluoroquinolone). This difference may be due to use of different methods of evaluation for determining the susceptibility. We determined the antimicrobial resistance by the microdilution method which is more sensitive than disk diffusion method [22]. The results from other studies from Malaysia and Spain showed lower resistance to trimethoprim/sulfamethoxazole and ciprofloxacin in urine samples from adults than this study which may be due to different patient population (adults versus pediatrics). Totally, the increasing resistance of* E. coli* to trimethoprim makes this drug less effective as empiric treatment of UTI [30].

The ESBL-producing* E. coli* isolates in our study showed high resistance to colistin which is in accordance with the studies reported by Benenson et al. [31] and Ku et al. [32]. Rapid increase in colistin resistance in* K. pneumoniae* strains was reported. Although some studies describe colistin activity against* Pseudomonas* and* Acinetobacter* isolates, the activity of colistin against ESBL-producing* E. coli* remains unclear [31, 33].

One of the most prominent and concerning findings in our study is the high resistance to broad spectrum antibiotics such as carbapenems which is in contrast with other studies that reported lower resistance (about 34%) to imipenem and meropenem in India [18], Malaysia [21], Columbia, Saudi Arabia [34], and Iran [24, 35, 36]. Although we found carbapenems as the most effective agent against the ESBL but the high rate of resistance, in comparison with other studies, is still very concerning. Recently, Alikhani et al. study in Iran showed 75% susceptibility among ESBL pathogens to carbapenems [20]. The main reason for large difference in the rate of resistance among different countries and different regions within the same country is due to the extensive use of broad spectrum antibiotics especially third generation cephalosporins and persistence of the resistant strains in health care facilities. Extensive usage of broad spectrum antibiotics specially third generation cephalosporins was reported by Salehifar et al. in our center. The rate of antibiotics consumption in our setting was significantly higher than other centers [37].

*TEM* was the most frequent resistant gene in ESBL-producing* E. coli* isolates in this study as it was also reported in several other studies [21, 24, 35].* GES* resistant gene was not found in ESBL-producing* E. coli* isolates which is in line with the low frequency of this gene in ESBL-producing* E. coli* strains [36].

Analysis of the genotypes, antimicrobial resistance pattern, and MIC of different antimicrobial agents for ESBL-producing* E. coli* isolates showed a significantly higher resistance to ceftriaxone, cefotaxime, and amikacin in* TEM* genotype negative group. The study by French et al. showed that the* SHV* producing strains of* E. coli* were resistant to all aminoglycosides but sensitive to ciprofloxacin [38] but in this study the* SHV* genotype positive isolates were significantly resistant to both aminoglycosides and ciprofloxacin.

About 82% and 50% of* CTX-M* producing strains were resistant to quinolones and aminoglycosides, respectively, which was higher compared to Edelstein et al. study (21%) [39] but it was lower than the Mendonça et al. study (93%) [40]. The resistance to cefotaxime in* CTX-M* producers in our study was higher than those reported.

## 5. Conclusion 

Although the* TEM* was found to be the most prevalent resistant gene, the prevalence of other resistant genes along with antimicrobial resistance is on the rise. Carbapenems were the most effective antibiotics against ESBl-producing* E. coli* in urinary tract infection in North of Iran. Considering the high prevalence of* SHV*, aminoglycosides and fluoroquinolones are not recommended for empiric therapy. The high rate of* SHV* and* VEB* transmission will result in increasing the resistance to third generation cephalosporins, aminoglycosides, and fluoroquinolones.

## Figures and Tables

**Table 1:** The sequences of primers and thermal condition used in PCR amplification.

Target genes	Primer used (5′-3′)	Thermal cycling condition	PCR product size
*TEM *	TAATCAGTGAGGCACCTATCTC GAGTATTCAACATTTCCGTGTC [18, 19]	94°C 3 min → 35 × [94°C 30 sec, 45°C 45 sec, 72°C 40 sec] → 72°C 7 min	800 bp

*CTX *	TTTGCGATGTGCAGTACCAGTAA CGATATCGTTGGTGGCATA [20]	94°C 5 min → 40 × [94°C 45 sec, 53.1°C 45 sec, 72°C 1 min] → 72°C 7 min	593 bp

*VEB *	CGACTTCCATTTCCCGATGC GGACTCTGCAACAAATACGC [9]	93°C 3 min → 40 × [93°C 1 min, 54.9°C 1 min, 72°C 1 min] → 72°C 7 min	585 bp

*SHV *	GGTTATGCGTTATATTCGCC TTAGCGTTGCCAGTGCTC [21]	1 cycle of 5 min at 96°C; 35 cycles of 1 min at 96°C, 1 min at 60°C, 1 min at 72°C; 1 cycle of 10 min at 72°C	867 bp

*GES *	ATGCGCTTCATTCACGCAC CTATTTGTCCGTGCTCAGG [22]	1 cycle of 5 min at 95°C; 30 cycles of 1 min at 95°C, 45 sec at 55°C, 1 min 30 sec at 72°C; 1 cycle of 8 min at 72°C	846 bp

**Table 2:** Percentage of antimicrobial susceptibility in ESBL-producing *E. coli* strains based on MIC results.

Antimicrobial agents	*R* (%)	*I* (%)	*S* (%)	CLSI breakpoints (*µ*g/mL)	
				*S*	*R*
Cephalosporins					
Cefepime	67	13	20	≤8	≥32
Cefixime	99	0	1	≤0.25	≥1
Ceftriaxone	28	42	30	≤8	≥64
Ceftazidime	19	26	55	≤16	≥32
Ceftizoxime	46	27	27	≤8	≥64
Cefotaxime	13	40	47	≤8	≥64
Carbapenems					
Imipenem	23	11	66	≤4	≥16
Meropenem	18	15	67	≤4	≥16
Aminoglycosides					
Amikacin	34	8	58	≤16	≥64
Gentamicin	37	12	51	≤4	≥16
Others					
Ciprofloxacin	76	0	24	≤1	≥4
Colistin	82	0	18	≤2	≥4
Trimethoprim/sulfamethoxazole	65	7	28	≤2/38	≥4/76
Piperacillin/tazobactam	20	38	42	≤16/4	≥128/4

*R*: resistance, *I*: intermediate, *S*: sensitive, CLSI: Clinical Laboratory Standards Institute, and ESBL: extended-spectrum beta-lactamase.

**Table 3:** Association between gene expression and antimicrobial nonsusceptibility in ESBL-producing *E. coli*.

Antimicrobial agents	*TEM *		*CTX *		*VEB *		*SHV *	
	Positive	Negative	Positive	Negative	Positive	Negative	Positive	Negative
Cephalosporins								
Cefepime	38 (77.6%)	42 (82.4%)	23 (82.1%)	57 (79.2%)	6 (75%)	74 (80.4%)	38 (86.4%)	42 (75%)
Cefixime	49 (100%)	50 (98%)	27 (96.4%)	72 (100%)	8 (100%)	91 (98.9%)	43 (97.7%)	56 (100%)
Ceftriaxone	29 (59.2%)	41 (80.4%)^**∗**^	22 (78.6%)	48 (66.7%)	4 (50%)	66 (71.7%)	33 (75%)	37 (66.7%)
Ceftazidime	22 (44.9%)	23 (45.1%)	11 (39.3%)	34 (47.2%)	6 (75%)^**∗**^	39 (42.4%)	17 (38.6%)	28 (50%)
Ceftizoxime	34 (69.4%)	39 (76.5%)	23 (82.1%)	50 (69.4%)	6 (75%)	67 (72.8%)	32 (12.7%)	41 (73.2%)
Cefotaxime	21 (42.9%)	32 (62.7%)^**∗**^	18 (64.3%)	35 (48.6%)	5 (62.5%)	48 (52.2%)	21 (47.7%)	32 (57.1%)
Carbapenems								
Imipenem	14 (28.6%)	20 (39.2%)	11 (39.3%)	23 (31.9%)	3 (37.5%)	31 (33.7%)	15 (34.1%)	19 (34%)
Meropenem	12 (24.5%)	21 (42.2%)	9 (32.1%)	24 (33.3%)	3 (37.5%)	30 (32.6%)	13 (29.5%)	20 (35.7%)
Aminoglycosides								
Amikacin	14 (28.6%)	28 (54.9%)^**∗**^	13 (46.4%)	29 (40.3%)	3 (37.5%)	39 (42.4%)	24 (54.5%)^**∗**^	18 (32%)
Gentamicin	21 (42.9%)	28 (54.9%)	14 (50%)	35 (48.6%)	5 (62.5%)	44 (47.8%)	27 (61.4%)^**∗**^	22 (39.3%)
Others								
Ciprofloxacin	34 (69.4%)	42 (82.4%)	21 (75%)	55 (76.4%)	5 (62.5%)	71 (77.2%)	39 (88.6%)^**∗**^	37 (66%)
Colistin	39 (79.6)	43 (84.3%)	23 (82.1%)	59 (81.9%)	6 (75%)	76 (82.6%)	38 (86.4%)	44 (78.6%)
TMP/SXT	34 (69.4%)	38 (74.5%)	21 (75%)	51 (70.8%)	4 (50%)	68 (73.9%)	29 (65.9%)	43 (76.8%)
Pip/TBZ	29 (59.2%)	29 (59.2%)	15 (53.6%)	43 (59.7%)	4 (50%)	4 (50%)	25 (56.8% )	33 (59%)

*R*: resistance, *I*: intermediate, *S*: sensitive, and ESBL: extended-spectrum beta-lactamase.

^∗^Significant differences (*P* < 0.05).
